# Liver Elastography as a Predictor of Esophageal Varices in Patients With Cirrhosis

**DOI:** 10.7759/cureus.18593

**Published:** 2021-10-08

**Authors:** Muhammad Danish, Hina Ismail, Rani Tulsi, Nasir Mehmood, Syed Muddasir Laeeq, Nasir Hassan Luck

**Affiliations:** 1 Department of Gastroenterology and Hepatology, Sindh Institute of Urology and Transplantation, Karachi, PAK; 2 Department of Gastroenterology, Sindh Institute of Urology and Transplantation, Karachi, PAK

**Keywords:** portal vein, liver stiffness, liver elastography, hepatitis, endoscopy, gastric varices, esophageal varices, cirrhosis

## Abstract

Introduction

Liver cirrhosis is an advanced consequence of a long-standing liver disease that can lead to portal hypertension which subsequently can manifest as life-threatening variceal bleeding. The present study aimed to determine liver stiffness by shear wave elastography (SWE) to predict esophageal varices (EV) in patients with chronic liver diseases.

Methodology

A prospective observational study was performed at the Department of Hepatogastroenterology, Sindh Institute of Urology and Transplantation, Karachi between November 2020 and July 2021. Individuals aged between 18 and 50 years, irrespective of gender, with diagnosed chronic liver disease >6 months were eligible to partake in the study. Patients with concomitant renal failure, severe ascites, severe life-threatening co-morbidities including congestive heart failure NYHA-III and IV, acute asthmatic attack, and recent myocardial infarction (MI) were excluded from the study. SWE was determined in all patients to measure liver stiffness. Esophagogastroduodenoscopy (EGD) was performed to visualize the esophageal varices. All findings were recorded. A 2 × 2 table was applied to determine the sensitivity, specificity, positive, and diagnostic accuracy for SWE by taking endoscopy as the definitive test.

Results

A total of 204 patients were included in the study. Mean age of 40.37 ± 15.20 years was observed. The mean liver size of patients was 12.38 ± 2.06 cm and the mean liver stiffness of patients was 19.97 ± 8.6. The sensitivity and specificity of liver elastography were 44.90% and 51.90%, respectively. Furthermore, the positive and negative predictive values were 53.00% and 99.39%, respectively. The diagnostic accuracy of the test was 51.86%.

Conclusion

Even though the diagnostic gold standard for the detection of varices is EGD, liver elastography provides a less invasive procedure to assess for varices in patients with cirrhotic liver disease. The present study concluded that liver elastography is a non-invasive and indirect valuable tool to predict the presence of esophageal varices with sensitivity and specificity of 44.90% and 51.90%, respectively.

## Introduction

Cirrhosis of the liver is an advanced complication of a long-standing liver disease arising from continuous exposure to injury [1]. It is marked by the progressive functional hepatic architecture replacement by non-functional fibrotic tissue and nodule formation [2]. Portal hypertension is an important complication of liver cirrhosis which results in the formation of portosystemic collateral circulation in the form of esophageal varices (EVs) [3]. The EVs are present in 40% to 85% of cirrhotics depending on the Child Turcotte Pugh (CTP) class [4]. Annually, 3-12% of patients with cirrhosis develop esophageal varices, and in 8-12% varices progress in size. Furthermore, the mortality rate due to variceal bleed ranges from 20% to 35% [5]. According to the American Association for the Study of Liver Diseases (AASLD), esophagogastroduodenoscopy (EGD) should be performed in all newly diagnosed cirrhotic [6].

Although EGD is the gold standard for esophageal varices diagnosis, the associated risk of complications is not negligible. Moreover, initial screening EGD is followed by subsequent surveillance depending on baseline findings and also after new-onset decompensation according to the European Association for the study of liver disease [7,8]. Apart from the invasiveness of EGD for diagnosis, it also imposes financial implications and psychological trauma to the patient which implies that an alternative non-invasive diagnostic parameter is identified.

Multiple studies have identified noninvasive predictors of EVs but have shown partial correlation with the presence of EV [9]. Liver stiffness has been linked with an increased risk of esophageal varices and could be used as a non-invasive technique to assess and detect varices. Transient elastography (TE) is based on the transmission of mechanical waves while shear wave elastography (SWE) depends upon the sound waves to determine the stiffness. Studies demonstrate that liver stiffness on the TE of greater than 20 kPa in addition to a platelet count of greater than 150 thousand per microlitre is associated with a significantly low rate of varices [9-11]. 

Nevertheless, TE has certain drawbacks, for instance, it does not give accurate results if the patient is obese, has ascites, or has limited intercostal spaces [10,11]. SWE has partly overcome these limitations and has shown higher success rates [12-15]. Liver stiffness measurement (LSM) by SWE in patients with cirrhosis to predict the esophageal varices, as suggested by studies, shows satisfying accuracy with sensitivity and specificity as high as 80% [16-18].

To the best of our knowledge, local literature is scarce regarding liver stiffness measurement for the prediction of esophageal varices. By performing this study, additional pretest probability regarding esophageal varices will be achieved and appropriate decision making may become feasible. The study aimed to measure liver stiffness by shear wave elastography to predict EV in patients with chronic liver diseases.

## Materials and methods

A prospective observational study was conducted at the Department of Hepatogastroenterology, Sindh Institute of Urology and Transplantation, Karachi between November 2020 and July 2021. A non-probability consecutive sampling technique was used for the recruitment of patients. The sample size was estimated using an electronic sample size calculator using the diagnostic accuracy indices from a previously published study [16]. By keeping the sensitivity and specificity around 85% with margins of error 13% and 95% confidence interval, a sample size of 200 was determined. Patients aged between 18 and 50 years, irrespective of gender, with diagnosed chronic liver disease, >6 months were eligible to partake in the study. Patients with concomitant renal failure, severe ascites, severe life-threatening co-morbidities including congestive heart failure NYHA-III and IV, acute asthmatic attack, and recent myocardial infarction (MI) were excluded from the study. Those with a history of variceal bleeding, hepatocellular carcinoma, or portal vein thrombosis were also excluded.

After the approval of this study from the institutional review board (SIUT-ERC-2020/A-236), all patients presenting to the Outpatients' Department of Hepatogastroenterology (GI-OPD), SIUT, Karachi diagnosed with liver cirrhosis, as per operational definition, were enrolled in the study. Written informed consent was obtained from all patients. Shear wave elastography was determined in all patients to measure liver stiffness. It was performed by a consultant radiologist; having more than three years post-fellowship experience; using US (TOSHIBA-apleo 50 Model MCM17545TS) and using elastography (TOSHIBA-apleo) by the same radiologist to minimize inter-observer variation.

EGD was performed for the presence or absence of esophageal varices. All findings were recorded. The patients were stratified on the basis of liver stiffness values ≥19.7 kPa and ≤19.6 kPa. The results were expressed in kilopascals (kPa) [17]. Ten successful measurements were carried out on each patient. The success rate was calculated as the number of validated measurements divided by the number of total measurements.

True-positive cases were defined as the presence of EV on endoscopy and liver stiffness value ≥19.7 kPa using Fibro scan while false positive was defined as the absence of EV on endoscopy and liver stiffness value ≥19.7 kPa using Fibro scan. True negatives were defined as the absence of EV on endoscopy and liver stiffness value ≤19.6 kPa using Fibro scan and false-negative cases were defined as the presence of EV on endoscopy and liver stiffness value ≤19.6 KPa using Fibro scan.

Statistical Package for the Social Sciences (SPSS 20.0, IBM Corp., Armonk, NY) was used for data analysis. Frequencies and percentages were computed for categorical variables like gender, cirrhosis etiology, and esophageal varices. Quantitative values like age, duration of cirrhosis, and liver stiffness value were presented as mean ± standard deviation. A 2 × 2 table was used to calculate sensitivity, specificity, positive predictive value, negative predictive value, and diagnostic accuracy for SWE by taking endoscopy as the gold standard. Effect modifiers like age, gender, and duration of cirrhosis were controlled through stratification. Post-stratification sensitivity, specificity, the positive and diagnostic accuracy of liver stiffness were calculated taking upper GI endoscopy as the gold standard by using a 2 × 2 table.

## Results

A total of 204 patients were included in the study. Mean age of 40.37 ± 15.20 years was observed with a body mass index of 23.19 ± 15.29 kg/m^2^. Patient characteristics are given in Table 1.

**Table 1:** Baseline characteristics of the patients in the study BMI: body mass index, HBV: hepatitis B virus, HCV: hepatitis C virus, HDV: hepatitis D virus, CTP: Child-Turcotte-Pugh.

Characteristics	n (%)
Age groups	
<18 years	24 (11.8%)
18–35 years	46 (22.5%)
36–50 years	72 (35.3%)
>50 years	62 (30.4%)
Gender	
Female	71 (34.8%)
Male	133 (65.2%)
BMI class	
Underweight	37 (18.1%)
Normal	114 (55.9%)
Overweight	25 (12.3%)
Obese	14 (6.9%)
Urban rural	
Urban	107 (52.5%)
Rural	97 (47.5%)
Ethnicity	
Sindhi	76 (37.3%)
Urdu	82 (40.2%)
Balochi	15 (7.4%)
Pashto	12 (5.9%)
Punjabi	11 (5.4%)
Other	8 (3.9%)
Education	
Primary	68 (33.3%)
Matric	23 (11.3%)
Intermediate	10 (4.9%)
Graduate	6 (2.9%)
None	97 (47.6%)
Etiology of cirrhosis	
HBV	37 (18.1%)
HCV	99 (48.5%)
HBV+HDV	23 (11.3%)
Autoimmune	13 (6.4%)
Other	32 (15.7%)
CTP class	
A	107 (52.5%)
B	72 (35.3%)
C	21 (10.3%)
Liver texture	
Altered	190 (93.1%)
Normal	14 (6.9%)
Liver margin	
Regular	67 (32.8%)
Slightly irregular	21 (10.3%)
Irregular	116 (56.9%)
Liver stiffness	
Yes	86 (42.2%)
No	100 (49%)

The mean liver size of patients was 12.38 ± 2.06 cm and the mean liver stiffness of patients was 19.97 ± 8.6. Mean albumin was 3.27 ± 0.72 and other laboratory values are mentioned in Table 2.

**Table 2:** Laboratory parameters of patients in the study CTP: Child-Turcotte-Pugh, MELD: model for end-stage liver disease, INR: international normalized ratio, APRI: aspartate aminotransferase to platelet ratio index, FIB4: fibrosis-4 index

Laboratory parameters	Mean ± SD
Hemoglobin	10.75 ± 2.1
Total leukocyte count	5.2 ± 2.4
Platelet	122.75 ± 80.42
Mean corpuscular value	81.13 ± 7.78
Prothrombin time	13.66 ± 2.99
INR	1.23 ± 0.21
Urea	36.70 ± 35.55
Creatinine	1.63 ± 2.88
Sodium	136.48 ± 11.87
APRI	2.45 ± 3.05
FIB4	5.29 ± 5.50
Total bilirubin	2.36 ± 5.69
Direct bilirubin	1.02 ± 2.63
Alkaline phosphatase	214.89 ± 185.73
Aspartate aminotransferase	74.39 ± 61.82
Alanine transaminase	49.75 ± 34.41
Gamma-glutamyltransferase	87.71 ± 105.08
Albumin	3.27 ± 0.72

Liver stiffness was significantly greater in patients with esophageal varices than those who did not have EVs (21.72 ± 8.88 vs. 18.44 ± 8.04; p=0.009). There were some missing cases, which were excluded from the final analysis. The sensitivity and specificity of liver elastography with a cut-off value of ≥19.7 kPa were 44.90% and 51.90%, respectively. Furthermore, the positive and negative predictive values were 53.00% and 99.39%, respectively. The diagnostic accuracy of the test was 51.86% (Table 3).

**Table 3:** Indices of the accuracy of liver stiffness as a predictor of esophageal varices

Sensitivity	Specificity	Positive predictive value	Negative predictive value	Diagnostic accuracy
44.90%	51.90%	53.00%	99.39%	51.86%

**Figure 1:**
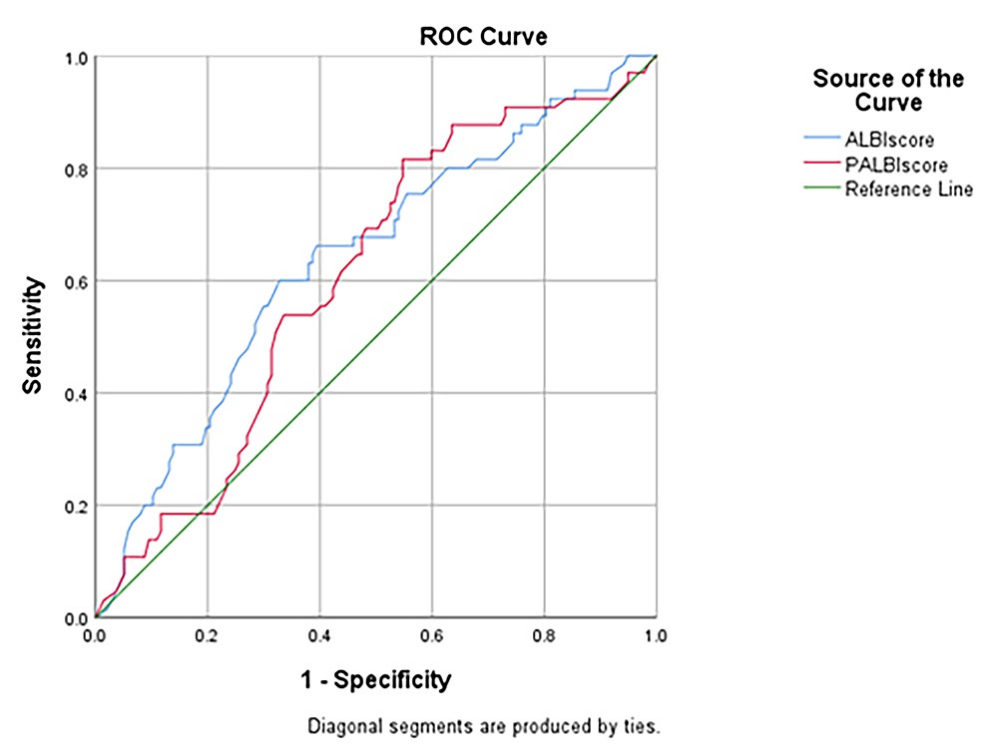
Receiver operating characteristics graph presenting area under the curve

The receiver operating characteristics (ROC) curve analysis was determined to assess the accuracy of liver elastography as a diagnostic tool for esophageal varices in patients with cirrhosis (Figure 1).

## Discussion

The aim of the study was to identify whether liver elastography was a good predictor of esophageal varices in patients with chronic liver diseases. There was a significant correlation between abnormal laboratory data and increased liver stiffness in our study. There was also a significant correlation between the degree of splenomegaly and the degree of liver stiffness at real-time shear wave elastography.

We also found that liver stiffness at real-time shear wave elastography was above 25 kPa in patients with varices. The sensitivity and specificity of liver elastography were 44.90% and 51.90%, respectively. This was similar to a study conducted by Muhammad et al. in which liver stiffness was found to be high in patients who had high-grade fibrosis (12.6 kPA) than in the control group (3.1 kPA) [19]. Real-time shear wave elastography was also done on the control group and the patients. The authors also found a correlation between increased liver stiffness and abnormal laboratory data which was similar to our study. Similarly, Berzigotti et al. and Tag-Adeen et al. discussed that the increase in liver stiffness resulted in an increased spleen size which led to a higher chance of finding varices [20,21]. We also found that liver stiffness predicted the occurrence of esophageal varices. Particularly, Tag-Adeen et al. found that spleen size greater than 15 cm is a good predictor of esophageal varices [21].

Giuffrè et al. in their study also discussed that the spleen stiffness probability model will help to find the number of patients who develop large or small varices by looking at the stiffness variation over time [22]. In our study, liver stiffness was found to have a sensitivity of 44.90% and 51.90%, respectively with the positive and negative predictive values being 53.00% and 99.39%, respectively. The diagnostic accuracy of the test was 51.86%. These findings were similar to the studies conducted by Zaki et al. and Hashim et al. who also found a link between liver stiffness and cirrhosis and portal hypertension which enables detection of gastric and esophageal varices via endoscopy [23,24].

Our study was not without limitations. Our sample size was small and thus more patients should have been included in the study. Follow-ups should have been done to find out the degree of the stiffness of the liver in the case of cirrhosis. The research could have been more extensive and could have been done on a larger scale. Invasive radiological methods for the assessment of liver stiffness should have been compared with non-invasive methods such as the fibroscan.

## Conclusions

Even though the diagnostic gold standard for the detection of varices is EGD, liver elastography provides a less invasive procedure to assess for varices in patients with cirrhotic liver disease. The present study concluded that liver elastography is a non-invasive and indirect valuable tool to predict the presence of esophageal varices with sensitivity and specificity of 44.90% and 51.90%, respectively.
